# Anti-Aging Effects of *Polygonatum* spp. Polysaccharides: Pharmacological Evidence, Molecular Mechanisms, and Structure–Activity Relationships

**DOI:** 10.3390/plants15213452

**Published:** 2026-11-05

**Authors:** Jianhong Xie, Jing Wang, Juan Xu, Lei Cheng, Yifan Zeng, Qingjun Shu, Yaping Wang, Baozhong Duan

**Affiliations:** 1 College of Pharmaceutical Science, Dali University, Dali 671000, China; 2 International Joint Laboratory for the Development and Utilization of Traditional Chinese Medicine Resources in Yunnan Province, Dali 671000, China

**Keywords:** *Polygonatum* spp. polysaccharides, anti-aging, molecular mechanisms, oxidative stress, gut microbiota, structure–activity relationships

## Abstract

*Polygonatum* species are medicinal and edible plants whose polysaccharides (PSPs) have attracted interest for their potential anti-aging effects. This review synthesizes evidence on the structural characteristics, pharmacological activities, molecular mechanisms, and translational prospects of PSPs. Relevant literature was retrieved from Web of Science and the China National Knowledge Infrastructure using terms related to *Polygonatum* polysaccharides, aging, structural characterization, and bioactivity. PSPs vary in molecular weight, monosaccharide composition, glycosidic linkages, and branching patterns. Studies using cells, *Caenorhabditis elegans*, and mammals report aging-related benefits associated with reduced oxidative stress and inflammation, modulation of gut microbiota and immune homeostasis, improved mitochondrial function, and metabolic regulation. Reported molecular changes were associated with Nrf2/HO-1, SIRT1/SIRT3, AMPK/PGC-1*α*, ERK/NF-κB, and DAF-16-related signaling. Although comparisons among PSP fractions suggest associations between structural features and biological activity, causal structure–activity relationships remain unconfirmed. Moreover, most evidence is preclinical, and differences in botanical sources, extraction methods, and polysaccharide characterization limit cross-study comparisons. Future research should prioritize standardized, structurally defined preparations, direct structure–activity testing, pharmacokinetics, long-term safety, and clinical validation to clarify the potential of PSPs for healthy aging.

## 1. Introduction

**Figure 1:**
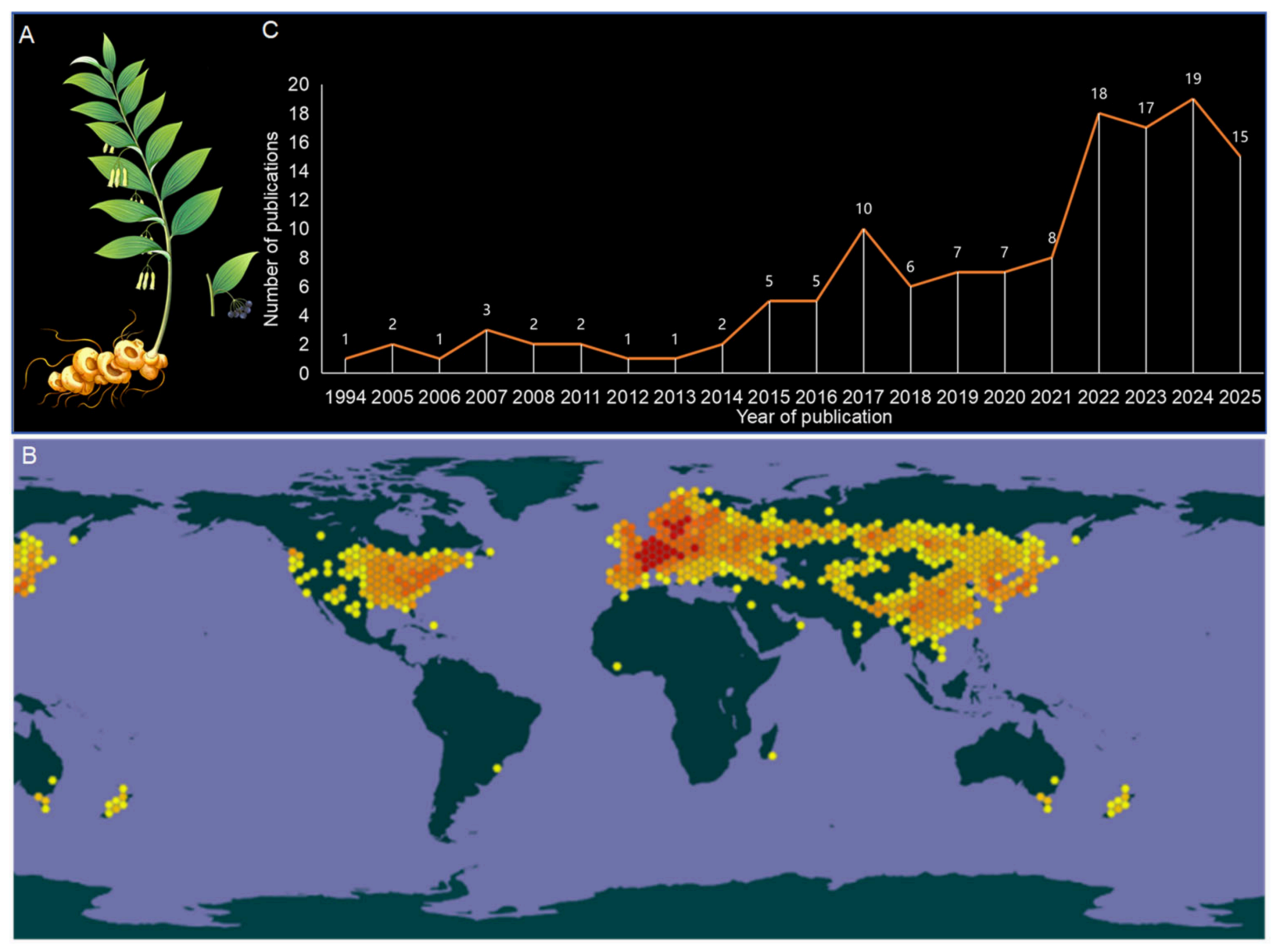
(**A**): The plant of *Polygonatum* spp.; (**B**): The distribution diagram of *Polygonatum* spp. species in the world (https://powo.science.kew.org/) (accessed on 27 October 2026); (**C**): The growth trend of the publications in the Web of Science database (https://www.webofscience.com/) (accessed on 27 October 2026) and China National Knowledge Infrastructure (https://oversea.cnki.net/index) (accessed on 27 October 2026) by topic-based searching: *Polygonatum*, polysaccharides, and anti-aging.

*Polygonatum* species have been traditionally used across Asia, including China, Japan, Korea, and India, to promote health and longevity [1]. These perennial herbs belong to the Asparagaceae family and comprise approximately 107 identified species (Figure 1B) [2]. In China, *Polygonatum* plants have long been regarded as longevity-promoting herbs in traditional Chinese medicine. Historical materia medica texts, such as the *Shen Nong Ben Cao Jing* and *Ben Cao Gang Mu*, document their use for enhancing vitality, delaying senescence, and promoting longevity [3]. In addition, in the Nu River Basin, *Polygonatum kingianum* (*P. kingianum*) is traditionally consumed to alleviate age-related musculoskeletal decline among the Lisu, Dulong, and Nu ethnic groups [4,5]. Collectively, these traditional uses provide a historical and ethnomedicinal rationale for investigating the biological basis of the potential anti-aging effects of *Polygonatum*.

Modern studies suggest that the anti-aging effects of *Polygonatum* are associated with diverse phytochemicals, including polysaccharides, saponins, terpenoids, alkaloids, and flavonoids [6]. Among these constituents, polysaccharides have emerged as important bioactive components with multiple aging-related pharmacological activities [7,8]. PSPs are structurally heterogeneous macromolecules, and variations in molecular weight, monosaccharide composition, glycosidic linkages, and branching patterns may contribute to differences in their biological activities. Accumulating evidence from cellular, *Caenorhabditis elegans*, and mammalian models suggests that PSPs modulate multiple aging-related processes, including oxidative stress, inflammation and immune homeostasis, mitochondrial dysfunction, gut microbial dysbiosis, and metabolic disturbances. These structural variations may also underlie differences in the anti-aging effects reported for PSPs from different *Polygonatum* species. The increasing number of publications on the anti-aging effects of *Polygonatum* spp. polysaccharides (PSPs) in recent years reflects growing scientific interest in their potential roles in aging-related health regulation (Figure 1C). Previous reviews have addressed the anti-aging effects of *Polygonatum* or specific pharmacological aspects of PSPs; however, the available literature has largely focused on individual species, particularly *Polygonatum sibiricum* (*P. sibiricum*), or broader biological activities rather than critically integrating anti-aging evidence across different *Polygonatum* species, experimental models, molecular mechanisms, and structural characteristics [3,9]. Studies of PSPs also vary considerably in the species investigated, structural characteristics reported, experimental models used, and molecular mechanisms examined. Consequently, the strength and consistency of the anti-aging evidence across different experimental systems remain insufficiently resolved, and the structural determinants underlying these effects are poorly defined. In particular, current structure–activity relationship (SAR) evidence is derived largely from comparisons among individual polysaccharide fractions rather than direct experimental verification of specific structural features. Moreover, pharmacokinetic and bioavailability data, long-term safety evaluations, standardized preparations, and clinical evidence remain insufficient, limiting the translation of experimental findings into therapeutic development. A comparison with six representative previous reviews is provided in Table 1 to further clarify the distinctive scope and contribution of the present review.

Accordingly, this review provides an aging-focused synthesis of PSPs by integrating traditional longevity-related uses with contemporary pharmacological evidence. Particular emphasis is placed on evaluating anti-aging evidence across cellular, *C. elegans*, and mammalian models; identifying convergent molecular mechanisms; and critically assessing emerging relationships between structural characteristics and biological activity. Safety, translational barriers, and potential implications for further development are also evaluated. By connecting structural characteristics with pharmacological outcomes and mechanistic evidence, this review aims to clarify the current strength and limitations of the evidence, identify key knowledge gaps, and provide a framework for the mechanistic and translational development of PSPs as potential anti-aging agents.

## 2. Literature Search Strategy

A literature search was conducted using “*Polygonatum*” combined with the keywords “polysaccharides,” “anti-aging,” “senescence,” “structural characterization,” “pharmacological activities,” and “molecular mechanisms” across Web of Science and China National Knowledge Infrastructure (CNKI), covering publications up to October 2026.

The retrieved records were screened according to predefined eligibility criteria using a PRISMA-informed approach. Inclusion criteria encompassed: (i) original research articles investigating polysaccharides derived from *Polygonatum* species; (ii) in vivo or in vitro studies examining anti-aging effects, aging-related biological activities, molecular mechanisms, or structural characteristics; and (iii) sufficient experimental or structural data to support the reported findings. Exclusion criteria were: (i) duplicate publications; (ii) studies unrelated to the scope of this review; and (iii) studies with ineligible designs or insufficient relevant experimental or structural data. Previous reviews, historical texts, patents, and studies involving multi-herb formulations were considered separately as contextual references.

During the eligibility phase, full-text articles were evaluated for relevance and methodological adequacy through a qualitative assessment considering model relevance, control design, outcome selection, characterization of the tested PSP preparations, and the extent of mechanistic validation. Pharmacological studies were assessed according to the appropriateness of experimental models, control groups, and aging-related outcome measures. Structural studies were evaluated based on the reliability of polysaccharide identification and characterization, including molecular weight, monosaccharide composition, and glycosidic linkage analysis. Furthermore, mechanistic studies were examined to distinguish biomarker associations from pharmacological interventions and genetic validation.

**Figure 2:**
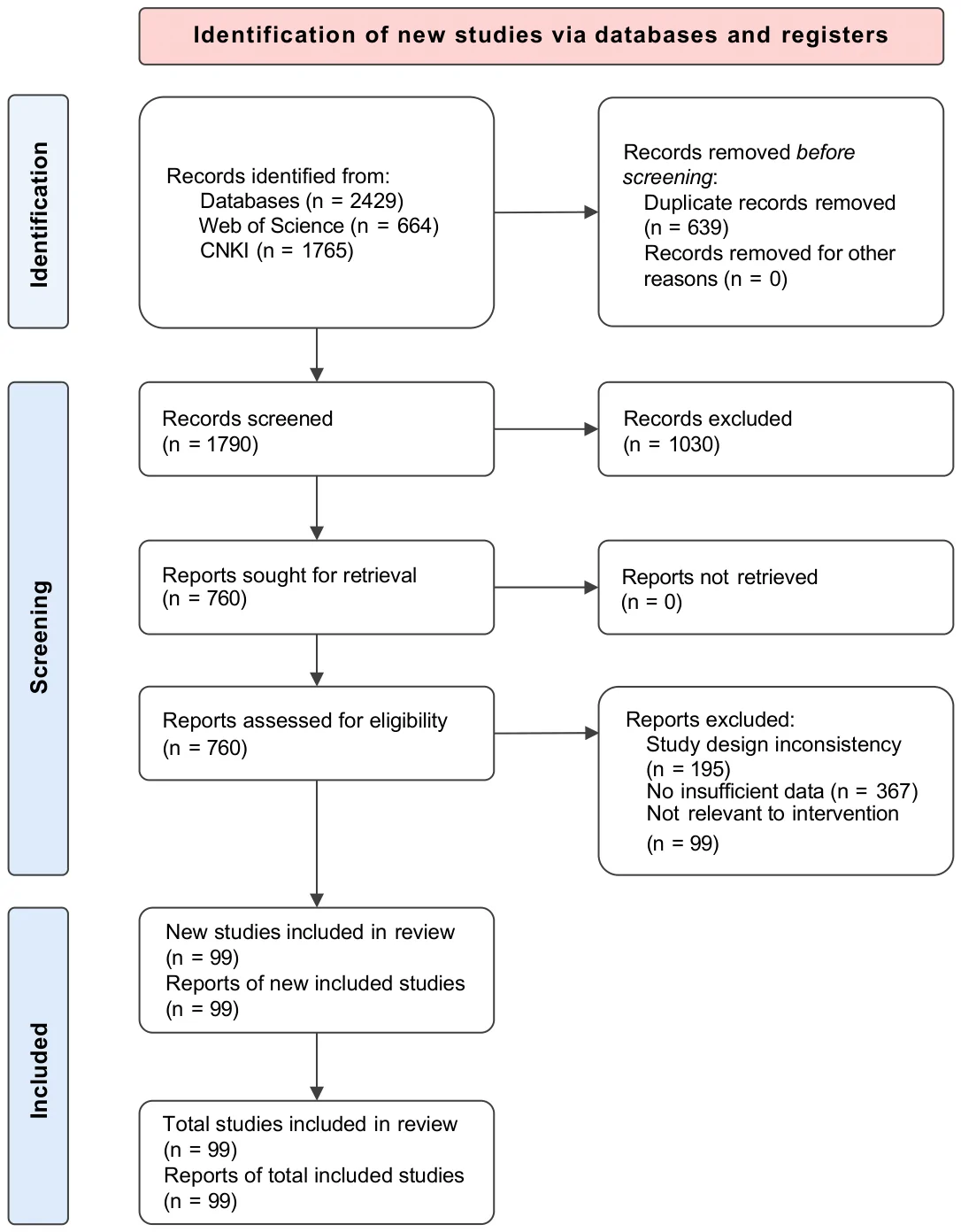
The PRISMA flowchart of the process for identifying candidate studies.

The database searches identified 2429 records, of which 639 duplicates were removed. Following title and abstract screening of 1790 records, 760 reports underwent full-text assessment. Of these, 661 were excluded because of ineligible study designs or publication types (*n* = 195), insufficient relevant data (*n* = 367), or lack of relevance to the review objectives (*n* = 99). Ultimately, 99 studies were deemed eligible for inclusion. The literature selection process is illustrated in Figure 2.

## 3. Traditional Basis for the Anti-Aging Use of *Polygonatum*

### 3.1. Historical Records

*Polygonatum* plants have long been recognized as longevity-promoting herbs in traditional Chinese medicine, as documented in numerous classical materia medica texts [3,10]. These sources consistently describe Polygonati Rhizoma as effective in extending lifespan, maintaining youthfulness, and delaying age-related decline. For instance, the earliest reference to its anti-aging properties appears in the *Ming Yi Bie Lu* (Supplementary Records of Famous Physicians), which states that long-term use can delay aging, suppress hunger, increase body weight, and improve cardiopulmonary function [11]. Similarly, the *Shen Nong Ben Cao Jing* (Divine Farmer’s Classic of Materia Medica) reports that Polygonati Rhizoma enhances complexion, boosts energy, and slows skin aging [12]. The Ben Cao Gang Mu (Compendium of Materia Medica) credits it with prolonging life and supporting the functions of the five internal organs [13]. Additional texts, such as the *Bo Wu Zhi* and *Zheng Lei Ben Cao*, further affirm its anti-aging properties, including hair darkening and tooth regeneration [14].

Beyond its anti-aging effects, Polygonati Rhizoma is traditionally believed to offer complementary health benefits that support long-term vitality. The *Dian Nan Ben Cao* (Materia Medica of Southern Yunnan) records its ability to strengthen muscles and bones, aid digestion, and alleviate fatigue [15]. The *Sheng Ji Zong Lu* (General Records of Sacred Formulas) identifies it as a tonic that reinforces vital energy, stabilizes essence, enhances blood circulation, and contributes to longevity [16]. These historical sources underscore *Polygonatum* plants as a central herb in anti-aging traditional remedies and provide valuable references for contemporary research.

### 3.2. Traditional and Contemporary Uses

The anti-aging potential of *Polygonatum* species is well recognized in both traditional and modern medical practices [17]. Numerous classical prescriptions emphasize the life-extending effects of Polygonati Rhizoma. For instance, the *Tai Ping Hui Min He Ji Ju Fang* includes a formula known as Huangjing Wan, which combines *Polygonatum* and *Codonopsis pilosula* to nourish the kidneys and essence, strengthen the spleen, and enhance blood circulation, thereby exerting anti-aging effects [18]. Similarly, the *Qi Xiao Liang Fang* records Erjing Wan, a remedy composed of *Polygonatum* and *Lycium barbarum*, recognized for tonifying qi and essence to support longevity [19]. Moreover, a *Polygonatum* Rhizome Paste from the *Qian Jin Yao Fang* (Essential Prescriptions Worth a Thousand Gold) is described as improving skin tone, darkening hair, and prolonging life [20]. Moreover, modern research also confirms the anti-aging properties of Polygonati Rhizoma. For instance, Jiu Zhuan Huangjing Wan has significantly delayed skin aging in mouse models [21,22]. Similarly, Erjing Wan has positively affected skin elasticity in aging mice [23]. Furthermore, Papaya-Huangjing Paste has exhibited strong antioxidant properties in aging mouse models [24]. As summarized in Table 2, the traditional and contemporary uses of Polygonati Rhizoma encompass diverse formulations and application forms aimed at alleviating aging-related manifestations and supporting longevity. Collectively, these traditional records and experimental studies provide complementary evidence supporting the long-standing association of Polygonati Rhizoma with longevity and aging-related health. However, its underlying mechanisms remain unclear and warrant further scientific investigation.

## 4. Sources of Anti-Aging PSPs

Multiple species of the *Polygonatum* genus contain polysaccharides with reported aging-related biological activities. These findings are summarized in Table 2 and Table 3. Among the 107 identified species, *P. sibiricum*, *P. cyrtonema*, *P. kingianum*, and *P. odoratum* have been investigated for their aging-related biological effects. For instance, *P. sibiricum* polysaccharides have been reported to attenuate cardiac injury and oxidative stress in a D-galactose-induced aging mouse model [8]. In addition, PSPs ameliorate skeletal muscle aging-related changes and mitochondrial dysfunction in experimental models, potentially involving PI3K/Akt/mTOR signaling [25]. In D-galactose-induced aging mice, PSPs have been shown to attenuate renal injury, accompanied by alterations in gut microbiota composition and metabolic profiles [26]. Moreover, PSPs have been reported to improve cognitive performance in D-galactose-induced aging mice, potentially involving the microbiota–gut–brain axis [27]. A neutral polysaccharide fraction (PSP-NP) has also been reported to alleviate intestinal dysfunction in naturally aged mice [28]. Additionally, a purified *P. sibiricum* polysaccharide fraction (PSP-1-1) was reported to extend the lifespan of *C. elegans* by approximately 29.9%, providing direct lifespan evidence in this model [29].

Polysaccharides derived from other *Polygonatum* species also show promising anti-aging effects. For instance, polysaccharides (PCHP) from *P. cyrtonema* extend lifespan and reduce biomarkers of aging, increasing the lifespan of *C. elegans* by approximately 15.78% [6,30]. Fermented polysaccharides (MFP/SFP) from *P. kingianum* have been reported to exhibit antioxidant and cardioprotective activities, including protective effects in H9C2 cells [31]. Furthermore, fermented polysaccharides from *P. odoratum* have been reported to modulate immune responses, antioxidant-related indicators, and intestinal microbiota in mice [32]. Taken together, polysaccharides from various *Polygonatum* species show promising aging-related biological activities; however, direct evidence of lifespan extension should be distinguished from protective effects observed in induced-aging, cellular, and other experimental models.

## 5. Structural Characterization

The biological activities of PSPs may be associated with their structural characteristics. As complex heteropolysaccharides, characterization of their molecular features is important for documenting structural diversity across PSP preparations. Key structural parameters, including molecular weight (*Mw*), monosaccharide composition, glycosidic linkages, branching patterns, and conformational features, vary considerably among PSP fractions without necessarily establishing their individual contributions to biological activity. The main structural characteristics of representative PSPs investigated in aging-related studies are summarized in Table 3.

### 5.1. Monosaccharide Composition

Monosaccharide composition and molar ratios have been associated with differences in the biological activities of polysaccharides [33,34]. Numerous studies have shown that the PSP fractions studied are heteropolysaccharides mainly composed of mannose (Man), rhamnose (Rha), glucose (Glc), galactose (Gal), fructose (Fru), arabinose (Ara), and galacturonic acid (GalA) in distinct molar ratios [35,36]. For instance, a polysaccharide fraction (PS2-4) extracted from fermented *P. kingianum* was structurally characterized and found to contain Glc, Fru, GalA, Gal, Xyl, Fuc, Ara, GlcA, and Man in a molar ratio of 69.80:18.18:4.03:2.72:1.74:1.99:0.42:0.67:0.45 [37]. Similarly, a homogeneous polysaccharide (PCP-1) from *P. cyrtonema* was characterized as containing Man, Glc, Gal, and Ara in a molar ratio of 1:2.67:0.25:0.089 [6]. Moreover, a neutral polysaccharide (PSP-NP) isolated from *P. sibiricum* was characterized as containing Gal, Man, and Glc in a molar ratio of 68.7:24.3:7.0 [28].

These studies show that PSP fractions examined in aging-related studies differ substantially in their monosaccharide profiles. However, the available evidence does not establish whether individual monosaccharides or specific molar ratios directly determine anti-aging activity. Comparisons among structurally defined PSP fractions are therefore needed to clarify the contribution of monosaccharide composition to their biological effects.

### 5.2. Molecular Weight

*Mw* is an important structural parameter that may influence the physicochemical and biological properties of natural polysaccharides [6]. PSP fractions investigated in aging-related models span a relatively broad *Mw* range, from approximately 2.7 to 650 kDa. For instance, a low-molecular-weight fraction (PSP-1-1) isolated from steamed *P. sibiricum* has been studied in *C. elegans*. Its molecular weight was determined to be 2.7 × 10^3^ Da through high-performance gel permeation chromatography analysis [29]. Furthermore, fermentation shifted the molecular-weight distribution of *P. kingianum* polysaccharides from 50–650 kDa to 2–100 kDa, as determined by SEC-MALS-RI. Among the resulting fractions, the 10–50 kDa PS2–4 fraction exhibited the strongest anti-aging activity in *C. elegans*, extending lifespan by 20.70% [37]. In another study, a polysaccharide (PCP-1) from *P. cyrtonema*, with a *Mw* of 3.35 × 10^3^ Da, was characterized and tested in *C. elegans* [6].

### 5.3. Chemical Structure

In addition to the monosaccharide composition and *Mw*, glycosidic linkages, backbone structures, and branching patterns have been characterized in several PSP preparations studied in aging-related models. For instance, the neutral polysaccharide fraction PSP-NP derived from *P. sibiricum* exhibits specific glycosidic linkages, including 1,4-*β*-D-Gal, 1,4,6-*β*-D-Gal, T-*α*-D-Man, 1,4-*α*-D-Glc, and T-*α*-D-Glc [28]. Additionally, a purified polysaccharide fraction (PSP-1-1) isolated from steamed *P. sibiricum* was found to have a predominantly linear 1,4-*β*-D-galactan backbone (→4)-*β*-D-Galp-(1→) with minor branching points at O-6 (→4,6)-*β*-Galp-(1→) [29]. These findings indicate considerable structural diversity among studied PSP fractions; however, the contribution of specific glycosidic linkages or branching patterns to their activity remains unclear and requires further investigation.

## 6. Anti-Aging Pharmacological Activities of PSPs

**Figure 3:**
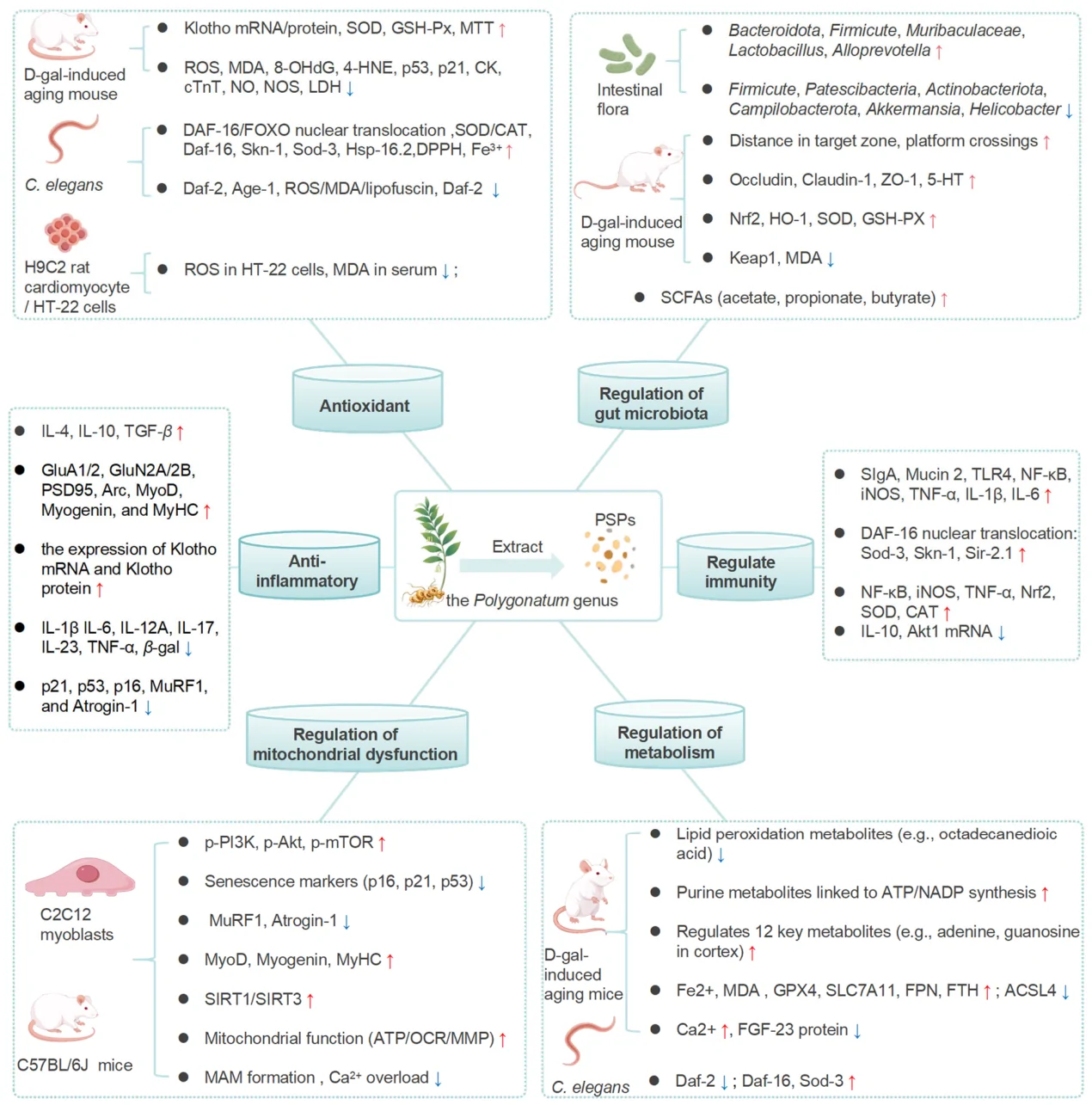
Anti-aging characteristics and molecular mechanisms of PSPs (created using the Figdraw online platform, https://www.figdraw.com/).

PSPs are important bioactive constituents of *Polygonatum* species and show activities relevant to aging through multiple pathways, including antioxidant and anti-inflammatory activities, modulation of gut microbiota, immune system regulation, and metabolic enhancement. The available evidence includes direct lifespan and healthspan outcomes in *C. elegans*, aging-associated biomarkers, findings from chemically induced aging models, age-related disease models, and cell-based stress or senescence models. However, these findings differ in their strength of evidence and do not necessarily demonstrate delayed organismal aging. Furthermore, changes in signaling pathway markers or gut microbiota composition alone cannot establish causal relationships without appropriate experimental interventions. Table 4 and Figure 3 summarize the corresponding findings and proposed mechanisms.

### 6.1. Antioxidant Effects

The antioxidant system functions by neutralizing reactive oxygen species (ROS) and preserving cellular homeostasis. Oxidative stress arises when ROS production exceeds clearance capacity, leading to excessive ROS accumulation that may accelerate the aging process [38,39]. Studies have reported antioxidant effects of PSPs in aging-related experimental models. For instance, oral administration of *P. sibiricum* polysaccharide (PSP) at 200 or 400 mg/kg/day for 60 days attenuated oxidative damage in D-gal-induced cardiac aging mice. Treatment was associated with lower myocardial levels of ROS, MDA, 4-HNE, and 8-OHdG and higher superoxide dismutase (SOD) activity [8]. Moreover, oral administration of *P. cyrtonema* polysaccharides (PCP-1) at an optimal concentration of 1 mg/mL significantly prolonged lifespan and reduced aging biomarkers, such as lipofuscin, ROS, and MDA in *C. elegans*. This effect was likely mediated by downregulating the IIS pathway and activating DAF-16/FOXO nuclear translocation, thereby enhancing antioxidant defenses [6]. Similarly, other *P. cyrtonema* polysaccharides have also been reported to improve antioxidant defenses [40]. Additionally, fermented *P. kingianum* polysaccharides showed antioxidant and cardioprotective activities in experimental assays and cell models [31]. In summary, PSPs display antioxidant activities relevant to aging-related stress.

### 6.2. Regulation of Gut Microbiota

Changes in the composition of gut microbiota are closely linked to the aging process of the host [41,42]. Studies have reported that PSP administration is associated with alterations in gut microbiota composition and aging-related outcomes. For instance, oral administration of *P. sibiricum* polysaccharides at 150–600 mg/kg/day alleviated cognitive impairment in D-gal-induced aging mice, accompanied by increased relative abundance of Bacteroidota and reduced relative abundance of Firmicutes, Actinobacteriota, and Campilobacterota. These changes were associated with altered purine and glycerophospholipid metabolism [27]. In another study, steamed *P. sibiricum* polysaccharide (PSP-1-1) at 100 μg/mL extended the lifespan of *C. elegans* by approximately 29.9%. Separate in vitro human gut microbiota fermentation experiments showed changes in microbial composition and increased short-chain fatty acids (SCFAs), while glycolysis-related genes, including *pfk-1.1*, were altered in the worm experiments [29].

Collectively, these studies suggest that gut microbiota and metabolic changes are associated with beneficial effects in aging-related and disease models [43,44]. However, changes in microbial abundance and SCFA levels do not establish causality. Further studies using microbiota-targeted interventions, appropriate dietary controls, and longitudinal sampling are needed to clarify their causal contributions.

### 6.3. Regulation of Mitochondrial Function

**Figure 4:**
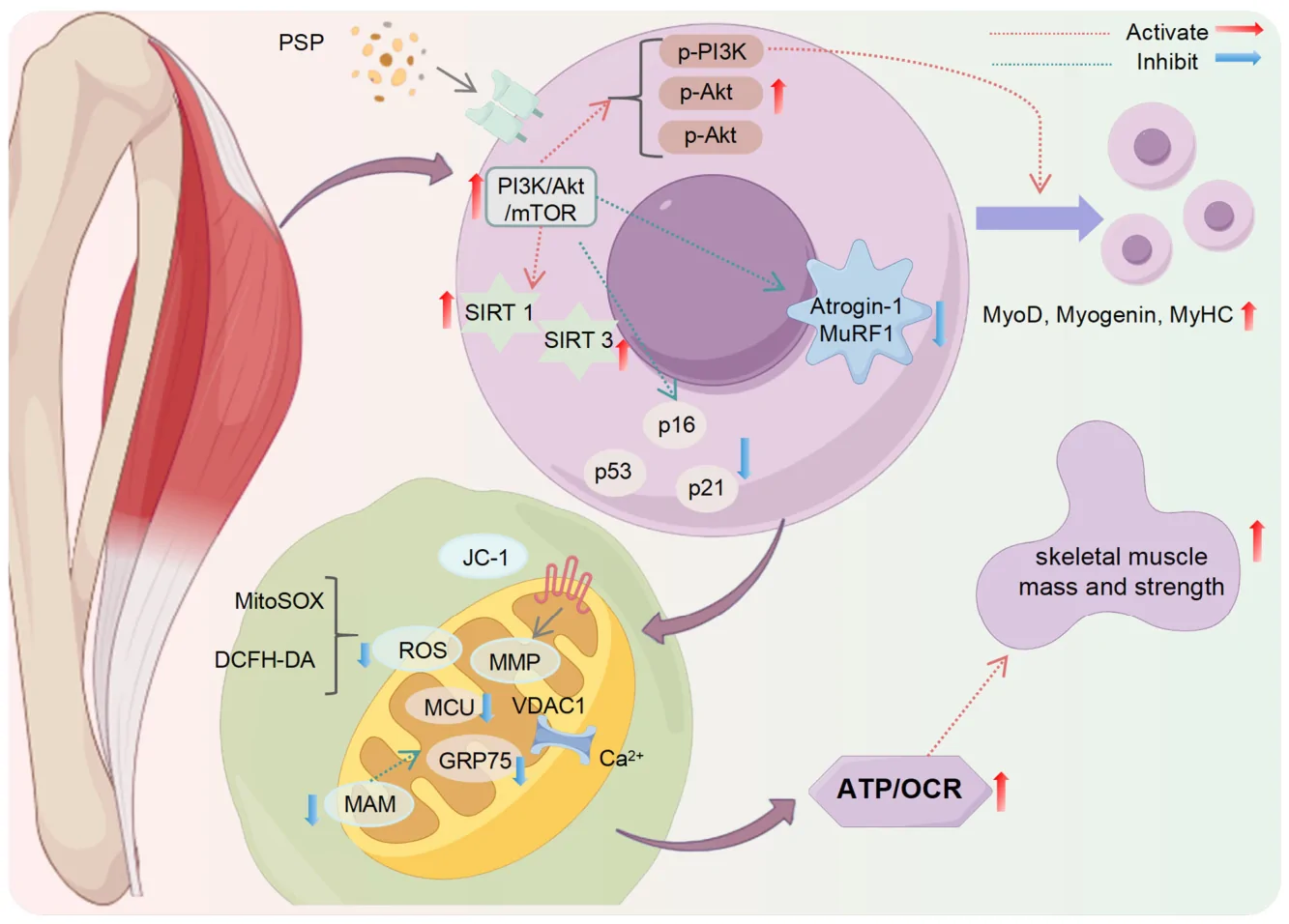
PSP-mediated amelioration of skeletal muscle aging (created using the Figdraw online platform, https://www.figdraw.com/).

Mitochondrial dysfunction reduces cellular energy production and increases free radical generation and is implicated in aging [43,44]. Recent studies suggest that PSPs may alleviate skeletal muscle dysfunction in experimental aging models by improving mitochondrial function and calcium homeostasis. Figure 4 illustrates proposed pathways associated with these findings. For instance, in D-gal-induced C2C12 cells, PSP treatment at 0.6–1.2 mg/mL altered PI3K/Akt/mTOR signaling and reduced senescence-related markers. At 50–100 mg/L, PSPs improved mitochondrial function and reduced mitochondria-associated membrane (MAM) formation and calcium overload. In vivo, PSPs at 60–120 mg/kg improved skeletal muscle outcomes in D-gal-induced aging mice, whereas treatment at 200 mg/kg/day improved mitochondrial function and muscle-related outcomes in naturally aged mice. These effects were accompanied by alterations in PI3K/Akt/mTOR signaling, SIRT1/SIRT3, reactive oxygen species (ROS) levels, and MAM-related proteins. However, differences in experimental models and treatment protocols limit direct comparisons across studies [25,45]. In summary, these studies support protective effects on skeletal muscle and mitochondrial dysfunction in aging-related models; however, their precise molecular targets and long-term efficacy require further investigation [46].

### 6.4. Regulation of Metabolism

Metabolic disorders are associated with aging and contribute to age-related diseases [47,48]. Studies suggest that PSPs can modulate metabolic abnormalities in experimental models. For instance, oral administration of PSP at 150–600 mg/kg/day attenuated kidney injury and altered metabolomic profiles in D-gal-induced aging mice. These metabolic alterations involved amino acid and lipid metabolism [26]. In another study, oral administration of PSP at 150–600 mg/kg/day improved cognitive outcomes in D-gal-induced aging mice and was associated with changes in purine, taurine/hypotaurine, glycerophospholipid, and amino acid metabolism [27]. Moreover, treatment with *P. kingianum* polysaccharides (PKPs) at 200 mg/kg/day improved fatigue-related outcomes in mice. These effects were accompanied by changes in energy metabolism and markers associated with the AMPK/PGC-1*α*/TFAM and Nrf2/HO-1/NQO1 signaling pathways [49]. Collectively, PSPs exhibit potential in ameliorating metabolic disorders associated with aging.

### 6.5. Immunomodulatory Effects

Immune function changes with age, and immune dysregulation is associated with aging [50,51]. Studies have reported immunomodulatory effects of PSPs in aging-related models. For instance, *P. sibiricum* neutral polysaccharide (PSP-NP) administered at 50–200 mg/kg/day improved intestinal function in naturally aged mice and was associated with changes in mucosal immune indicators, including secretory IgA and Mucin 2 [28]. Furthermore, fermented polysaccharides from *P. odoratum* improved immune-related outcomes and altered antioxidant and gut microbiota measures in mice [32]. Collectively, these findings suggest that PSPs may have beneficial effects on age-related immune dysfunction. However, further studies are needed to determine whether these immunomodulatory effects delay organismal aging.

### 6.6. Anti-Inflammatory Effects

Chronic inflammation is a contributor to aging and many age-related diseases [52,53]. PSPs have been reported to reduce inflammatory measures in aging-related or disease models. For instance, oral administration of *P. sibiricum* neutral polysaccharide (PSP-NP) at 50–200 mg/kg/day for 6 weeks improved intestinal function in naturally aged mice, accompanied by higher anti-inflammatory cytokines (IL-4, IL-10, TGF-*β*) and lower pro-inflammatory cytokines (IL-6, IL-1*β*, TNF-*α*) [28]. Moreover, *P. sibiricum* polysaccharide reduced inflammation and oxidative-stress-related measures in a depression-like behavior model [54]. Additionally, *P. sibiricum* polysaccharide attenuated IL-1*β*-induced inflammatory responses in cultured human chondrocytes, reducing NO, PGE2, iNOS, COX-2, TNF-*α*, and IL-6; these findings provide in vitro evidence relevant to osteoarthritis-associated inflammation [55]. Collectively, these findings suggest that PSPs may alleviate aging-related inflammatory responses. However, further studies are needed to determine whether these anti-inflammatory effects can delay organismal aging.

### 6.7. Other Pharmacological Activities

In addition to the activities described above, PSPs exhibit other pharmacological effects relevant to aging-associated conditions. For example, a purified *P. sibiricum* polysaccharide fraction (PSP50-2-2) promoted osteoblast differentiation and mineralization in vitro at the tested micromolar concentrations, providing evidence of osteogenic activity [46]. Furthermore, *P. cyrtonema* polysaccharide attenuated dopaminergic neuronal injury and Parkinsonian manifestations in an MPTP-induced mouse model, accompanied by changes in oxidative- and endoplasmic-reticulum-stress-related markers, including Nrf2, Bcl-2, Bax, and CHOP [56]. In another study, *P. sibiricum* polysaccharide (PSP-1) at 30 mg/kg/day improved cognitive performance and reduced amyloid-related pathology in 5 × FAD transgenic Alzheimer’s disease mice, an age-related disease model rather than a normal aging model [57].

Additionally, oral administration of *P. sibiricum* polysaccharide (PSP) at 200–400 mg/kg/day for 60 days improved brain-related outcomes in D-gal-induced aging mice, accompanied by changes in circRNA-associated ceRNA networks and genes involved in synaptic plasticity and Wnt signaling [58]. Moreover, *Polygonatum* polysaccharide at 400 mg/kg/day for 12 weeks improved learning and memory in D-gal-induced aging rats, with accompanying changes in Nrf2 and ferroptosis-related markers including ACSL4, GPX4, and SLC7A11 [59]. Collectively, these findings suggest that PSPs may protect against aging-related conditions. However, evidence from disease models and chemically induced aging models alone cannot establish that PSPs delay organismal aging.

Overall, evidence for the anti-aging effects of PSPs remains preliminary and varies across experimental models. While studies in *C. elegans* demonstrate lifespan extension, improvements in cellular and animal biomarkers do not necessarily indicate delayed aging. Moreover, variations in PSP preparations and experimental designs limit cross-study comparisons and reproducibility. Mechanistic evidence also remains insufficient to establish causality. Future studies should prioritize standardized preparations, rigorous experimental designs, and independent validation.

## 7. Putative Structure–Bioactivity Associations

The anti-aging activity of plant polysaccharides may be influenced by their structural characteristics [60,61]. Molecular weight, monosaccharide composition, glycosidic linkages, and branching patterns have been associated with variations in the biological activities of polysaccharides [62,63]. For PSPs, however, most comparisons involve fractions that differ simultaneously in botanical origin, processing, extraction conditions, molecular-weight distribution, composition, and potentially impurity profiles. Accordingly, the relationships discussed below should be considered potential associations rather than established causal structure–activity relationships (SARs).

### 7.1. Monosaccharide Composition and Bioactivity

Previous studies of plant polysaccharides have explored potential associations between monosaccharide composition and aging-related outcomes [62,64]. For PSPs, there is insufficient evidence that a high glucose proportion independently confers stronger anti-aging activity. For instance, the homogeneous polysaccharide PCP-1, consisting of Man, Glc, Gal, and Ara, extended lifespan in *C. elegans*. Its monosaccharide composition differed from that of the unfractionated PCP, alongside differences in anti-aging activity [6]. Moreover, fermentation has been shown to significantly modify the monosaccharide composition of polysaccharides derived from *P. kingianum* polysaccharides (PKPS), alongside changes in aging-related outcomes. Notably, the glucose content in the tested PS2-4 fraction isolated from fermented PKPS increased to 69.80%. PS2-4 extended the lifespan of *C. elegans* by 20.70%, compared with 9.65% for the unfermented preparation; however, fermentation altered multiple properties beyond glucose content [37].

Additionally, a low-molecular-weight polysaccharide (PSP-1-1) with Gal as its predominant monosaccharide was isolated from steamed *P. sibiricum* and was reported to extend the lifespan of *C. elegans* [29]. Taken together, these findings do not establish a relationship between glucose content and anti-aging activity. Variations in molecular weight, glycosidic linkages, and other structural features make it difficult to isolate the contribution of glucose content. Further studies using polysaccharide fractions with different monosaccharide compositions but comparable structural characteristics are needed to clarify this relationship.

### 7.2. Molecular Weight and Bioactivity

*Mw* is another structural parameter that has been associated with the biological activity of PSPs [37,65]. Some PSP fractions with relatively low or moderate *Mw* have shown lifespan-related effects, but no independent Mw–activity trend has been established. For instance, three polysaccharides (PCP, PKP, PSP) were extracted from *P. cyrtonema*, *P. kingianum*, and *P. sibiricum*, respectively, and displayed distinct molecular weight profiles of 8.5, 8.7, and 10.0 kDa. Among these, PCP, with the lowest *Mw*, exhibited the highest antioxidant activity in the reported assays [66]. In the same study, the low-molecular-weight polysaccharide PSP-1-1 (2.7 kDa) at 100 μg/mL increased the PT50 of A*β*-induced paralysis in *C. elegans* by 51.42%, whereas PSP-2-1 showed a 36.33% increase at 300 μg/mL [29]. However, because the molecular weight of PSP-2-1 was not reported, the observed differences in activity cannot be directly attributed to molecular weight. Fermentation significantly reduced the predominant *Mw* distribution of PKPS from 50–650 kDa to 2–100 kDa, alongside differences in aging-related outcomes. Among the fractions, PS2-4 (10–50 kDa) demonstrated the most potent lifespan-extending effect, extending the lifespan of *C. elegans* by 20.70% relative to the control group, compared with the 9.65% increase reported for the unfermented preparation (PS1). In vitro, studies revealed that lower *Mw* fractions (PS2-2, *Mw* < 100 kDa and PS2-4) showed differences in antioxidant assays and cellular senescence-related outcomes in human lung fibroblasts [37].

These comparisons suggest possible *Mw*-related differences in biological activity but do not establish that *Mw* reduction directly enhances anti-aging activity. Moreover, Mw changes often accompany changes in monosaccharide composition and other structural properties. Thus, the independent contribution of *Mw* to bioactivity remains unclear, and current evidence does not support a universally optimal *Mw* range for anti-aging PSPs.

### 7.3. Glycosidic Linkages, Branching, and Bioactivity

Glycosidic linkages and branching patterns may also contribute to the biological activities of polysaccharides [67,68]. Several PSP preparations investigated in aging-related models have well-characterized glycosidic linkages and branching patterns, although direct evidence associating specific structural features with anti-aging effects remains limited. For instance, PSP-NP, containing various galactose-, mannose-, and glucose-based linkages, has been reported to improve intestinal barrier function and gut microbial homeostasis in aged mice [28]. Because its structural motifs were not individually manipulated, this co-occurrence does not demonstrate a structure–function relationship, and the contribution of individual linkages, including 1,4,6-*β*-D-Gal, has not been independently established. Similarly, PSP-1-1, which has a galactan-rich backbone with limited branching, extended *C. elegans* lifespan [29]. At present, however, the available evidence does not establish whether its galactan backbone or branching pattern directly accounts for this activity.

Overall, glycosidic linkages and branching patterns may contribute to PSP bioactivity, but specific structural motifs have not been established as determinants of anti-aging effects. Stronger evidence requires matched preparations, controlled depolymerization or enzymatic modification, and direct testing of the resulting fractions under comparable conditions.

## 8. Potential Applications

**Figure 5:**
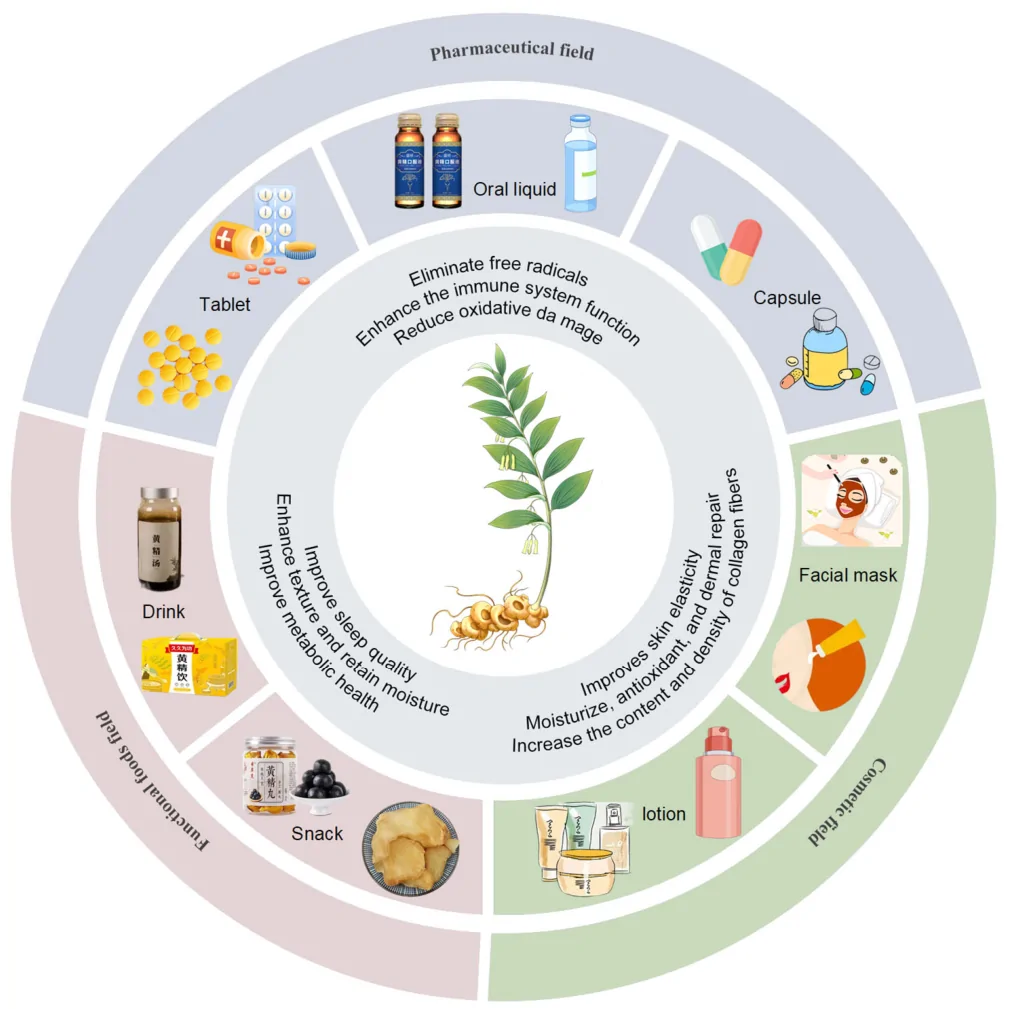
Application of PSPs in anti-aging (created using the Figdraw online platform, https://www.figdraw.com/).

PSPs have attracted interest for potential applications in aging-related health products due to their reported biological activities [69]. Their antioxidant, gut microbiota-modulating, metabolic, immunomodulatory, and anti-inflammatory activities support their exploration in pharmaceutical, food, and cosmetic products (Figure 5). However, translational evidence varies according to material type and evidence level. Purified or standardized PSP preparations are most relevant for evaluating PSP-specific effects, whereas the effects of crude *Polygonatum* extracts, processed rhizomes, and multi-herb formulations cannot be attributed specifically to PSPs. Traditional-use evidence provides historical context, whereas commercial products and patents reflect product development rather than established clinical efficacy. Accordingly, evidence from cellular and animal studies remains preclinical, and clinical efficacy requires validation in human studies involving adequately characterized PSP preparations.

### 8.1. Pharmaceuticals

Pharmacological interventions targeting aging generally aim to maintain physiological function or reduce the burden of aging-related disorders [70,71]. PSPs have been investigated in several aging-related conditions because of their antioxidant, metabolic, neuroprotective, and other pharmacological activities [72]. Experimental studies have reported beneficial effects of PSPs in models related to osteoporosis, Parkinson’s disease, Alzheimer’s disease, and age-related cognitive decline [46,56,57], suggesting possible pharmaceutical relevance rather than established therapeutic efficacy. PSP-containing tablets have also been proposed for anti-aging applications [73]. Additionally, PSP-containing oral liquids have been investigated for their effects on oxidative stress and physiological function [74]. In addition, PSPs have been formulated into oral capsules and evaluated for age-related oligoasthenospermia, with AMPK-mediated mitophagy proposed as a possible mechanism [75].

These studies indicate that PSPs have been explored in several oral dosage forms and aging-related conditions. Nevertheless, evidence supporting their clinical efficacy remains limited, and the available findings should not yet be interpreted as demonstrating established treatment effects. Further pharmacological and clinical evaluation is needed to define their therapeutic potential, effective doses, and long-term safety.

### 8.2. Cosmetics

Anti-aging cosmetics are designed to reduce or delay visible signs of skin aging, including dryness, wrinkles, loss of elasticity, and sagging [76]. PSPs have attracted attention as potential cosmetic ingredients because of their moisturizing, antioxidant, and skin-protective properties. For instance, PSP-containing formulations have been reported to produce an immediate moisturizing effect, with skin moisture reaching 62.26 C.U. within 30 min [77,78]. Such moisturizing properties may be relevant to maintaining skin hydration and elasticity, although they do not by themselves demonstrate an anti-aging effect.

Moreover, oxidative stress and lipid peroxidation contribute to skin barrier impairment and skin aging. PSPs (PCP) have been reported to increase SOD activity and reduce MDA levels, suggesting a protective effect against oxidative damage [79]. PSPs have also been associated with changes in collagen-related parameters and inhibition of tyrosinase activity and melanin formation [79,80]. These findings support the potential use of PSPs as multifunctional natural ingredients in cosmetic formulations. However, evidence directly demonstrating long-term improvements in clinically relevant skin-aging outcomes remains limited, and further studies are needed to establish their efficacy and safety in standardized cosmetic formulations.

### 8.3. Functional Foods

PSPs have also been incorporated into food and health-related products, including beverages, specialized foods, and biscuits, because of their reported biological activities [81,82]. For instance, *Polygonatum*-containing beverages have been proposed for anti-aging and sleep-related applications [83]. PSPs may additionally contribute to food texture and moisture retention, properties that may facilitate their incorporation into functional food formulations [84].

The potential value of PSPs in functional foods is also supported by their effects on gut microbial and metabolic homeostasis. Studies have reported modulation of beneficial gut microorganisms and increased production of short-chain fatty acids (SCFAs), together with improvements in metabolic parameters associated with obesity and other aging-related disturbances [85,86]. Polygonati Rhizoma has also been used in food products such as candied products and yogurt, although the biological effects of these products cannot necessarily be attributed solely to their polysaccharide content [20].

Overall, PSPs show potential as functional food ingredients because they combine reported biological activities with useful physicochemical properties. However, evidence linking PSP-containing foods directly to anti-aging outcomes in humans remains limited. Further work should clarify the relationship between structurally characterized PSPs, food formulations, and aging-related health outcomes.

## 9. Safety and Toxicity

Numerous studies have investigated the chemical composition and biological effects of PSPs; however, evidence specifically addressing their safety and toxicity remains limited. Dietary polysaccharides from *P. sibiricum* have been reported to exhibit low toxicity in available experimental evaluations [82]. For comparison, a 10-week toxicological study of *P. sibiricum* ethanol extract reported no apparent adverse effects at doses of up to 2000 mg/kg under the tested conditions [87]. Safety evaluations of processed *Polygonatum* materials, including those subjected to repeated steaming and drying, have also reported no significant toxicity under the tested conditions [88,89].

However, evidence regarding chronic toxicity, dose-dependent adverse effects, and the safety of long-term PSP administration remains insufficient. Potential drug interactions and contamination risks, including microbial contamination, heavy metals, and pesticide residues, should also be systematically evaluated. Given that anti-aging interventions may involve prolonged consumption, further toxicological and pharmacokinetic studies of well-characterized PSP preparations are essential to establish their safety profiles and appropriate dosage ranges before their broader application in pharmaceuticals and functional foods.

## 10. Summary and Prospect

This review integrates traditional uses, botanical sources, structural characteristics, anti-aging activities and mechanisms, SARs, safety, and potential applications of PSPs from an aging-focused perspective. Available evidence shows that PSPs act on multiple biological processes associated with aging, including oxidative stress, inflammation and immune homeostasis, gut microbiota, mitochondrial function, and metabolic homeostasis. PSPs with reported anti-aging activity are structurally diverse heteropolysaccharides, with *Mw* values ranging from approximately 2.7 to 650 kDa and considerable variation in monosaccharide composition, glycosidic linkages, and branching patterns. Comparisons among individual PSP fractions suggest possible associations between these structural characteristics and anti-aging activity; however, definitive causal SARs have not yet been established. PSPs have also been explored for pharmaceutical, cosmetic, and functional food applications, although most evidence supporting these uses remains experimental or preclinical. Taken together, current studies support the anti-aging potential of PSPs, but several important gaps need to be addressed before their biological effects can be translated into standardized and evidence-based applications.

(i) Most structural studies of PSPs have focused on monosaccharide composition, molecular weight, and glycosidic linkages, whereas higher-order structural information remains relatively limited. Spatial conformation and three-dimensional organization may influence biological activity, but their contribution to the anti-aging effects of PSPs remains unclear. More detailed structural characterization, combined with comparative biological evaluation, is needed to determine which structural features are consistently associated with specific anti-aging effects and to strengthen current SAR evidence.
(ii) Current evidence for the anti-aging activity of PSPs is derived predominantly from cellular, *C. elegans*, and animal models. These studies provide important mechanistic evidence but cannot establish efficacy in humans. Clinical studies are therefore needed to determine whether the effects observed in experimental models translate to aging-related outcomes in humans and to establish appropriate doses and durations of administration.
(iii) Safety data specific to purified or structurally characterized PSPs remain limited. Although available studies on PSPs, *Polygonatum* extracts, and processed materials generally report low toxicity under the tested conditions, evidence from crude extracts or whole plant materials cannot be directly extrapolated to individual PSP fractions. Long-term toxicity and dose-dependent adverse effects should therefore be evaluated alongside efficacy, particularly because anti-aging applications may involve prolonged consumption.
(iv) PSPs obtained from different *Polygonatum* species show considerable structural variation, which may contribute to differences in their biological activities. Processing can further alter their structural characteristics and associated activities. Such variability complicates comparison across studies and presents a challenge for consistent quality evaluation. Greater attention should therefore be given to linking botanical origin and well-defined structural characteristics with biological activity, together with the development of appropriate polysaccharide fingerprints or other quality-control approaches.
(v) PSPs have been investigated or incorporated into oral preparations, functional foods, and cosmetic formulations, but evidence supporting their anti-aging efficacy varies considerably among these applications. In particular, the transition from experimental biological activity to demonstrated efficacy in standardized products remains insufficient. Future product development should therefore be based on structurally characterized PSPs, reproducible quality specifications, appropriate safety assessment, and direct evaluation of aging-related outcomes rather than extrapolation from general antioxidant or health-promoting effects.

In conclusion, PSPs represent structurally diverse natural polysaccharides with multiple biological activities relevant to aging. Current evidence supports associations among their structural characteristics, modulation of aging-related biological processes, and beneficial outcomes in experimental models, but the causal relationships and clinical relevance remain incompletely defined. Integrating structural characterization with mechanistic, safety, and translational evidence will be important for determining the role of PSPs in future strategies for healthy aging.

**Table 1:** Comparison of the scope, evidence coverage, and distinctive contributions of previous reviews and the present review.

Year	Species	Scope	Structural Analysis	Aging Evidence	Mechanistic Evidence	Safety/Clinical Evidence	Refs
2024	*P. sibiricum*, *P. kingianum*, and *P. cyrtonema*	Polygonati Rhizoma; multiple constituent	Extraction and polysaccharide structures, alongside saponin/flavonoid biosynthesis	Substantial organ-centered synthesis of aging and age-related dysfunction	Organ-protective pathways discussed; no explicit intervention-based evidence hierarchy	Traditional use and processing-related irritation; no clinical anti-aging efficacy synthesis	[35]
2025	*P. sibiricum*	Polysaccharides: extraction, structure, bioactivities, SARs, and applications	Detailed composition, linkages, molecular weight and structure–activity relationships	Anti-aging examples within broader bioactivities; models not comparatively graded	Multiple functional pathways; causal strength not consistently stratified	Dedicated safety discussion; food applications; clinical anti-aging efficacy not established	[90]
2025	*P. sibiricum*	Polysaccharides: isolation, structure, health benefits, and applications	Isolation, structural diversity, modification and bioactivity links	Anti-aging among diverse health benefits; selected model outcomes	General activity mechanisms; limited comparison of genetic vs. associative evidence	Food and drug-carrier potential; clinical trials proposed as a future need	[91]
2025	*P. sibiricum*	Dietary polysaccharides: preparation, structure, functions, and food applications	Preparation, composition, structural features and structure–function links	Aging-related bioactivities mentioned; no dedicated model-level evaluation	Broad functional explanations; limited aging-specific causal assessment	Food products and general safety considerations; clinical validation identified as needed	[82]
2025	*P. sibiricum*, *P. kingianum*, and *P. cyrtonema*	Polygonati Rhizoma polysaccharides: extraction, structure, bioactivities, and drug-carrier applications	Extraction, 53 polysaccharide fractions, structural features and modifications	Anti-aging summarized among multiple pharmacological effects	Selected pharmacological pathways; no explicit hierarchy for aging mechanisms	Drug-carrier applications; safety and clinical anti-aging evidence not systematically assessed	[92]
2026	Multiple *Polygonatum* species	Polysaccharides: extraction, processing, pharmacological activities, and applications	Polysaccharide classes and processing-dependent structure–activity changes	Anti-aging among broader activities; limited endpoint-specific comparisons	Oxidative, inflammatory and other pathways reviewed without formal causal grading	Product development across sectors; clinical efficacy evidence remains limited	[93]
2026	Multiple *Polygonatum* species	Anti-aging effects of polysaccharides: evidence, mechanisms, and putative SARs	Relates structural heterogeneity and fractions to aging-related outcomes	Distinguishes lifespan, naturally aged, induced-aging and cellular-senescence evidence	Separates pathway association, pharmacological perturbation and genetic validation	Critically discusses standardization, safety-data gaps and absence of robust anti-aging clinical evidence	-

**Table 2:** Traditional uses and selected contemporary evidence for *Polygonatum*-containing preparations relevant to longevity and aging-associated outcomes.

Source/Study	Period/Study Design	*Polygonatum*-Containing Material	Preparation/Dose/Regimen	Model/Context	Outcomes/Indicators	Main Recorded Uses/Findings	Refs
*Letter from Ji Kang to Shan Ju Yuan*	Three Kingdoms period; historical record	*Atractylodes* and *Polygonatum*	Ingestion; exact preparation and dose not specified	Historical Daoist longevity practice	Longevity and physical vitality	Traditional statement associating ingestion of *Atractylodes* and *Polygonatum* with longevity	[3]
*Shen Nong Ben Cao Jing*	Han-era materia medica text; traditional attribution	*Polygonatum* rhizome	Long-term consumption described; dose and preparation not specified	Classical materia medica	Longevity, vitality, and hunger	Long-term use is traditionally associated with a lighter body, remaining youthful, and longevity	[12,20]
*Ming Yi Bie Lu*	Classical materia medica; early medieval text	*Polygonatum* rhizome	Dose and preparation not specified in the cited passage	Classical materia medica	Qi deficiency, wind-dampness, and visceral function	Traditional uses of replenishing middle qi, dispelling wind-dampness, and harmonizing the five viscera	[11,20]
*Bo Wu Zhi*	Western Jin period; historical record	*Polygonatum* rhizome	Ingestion; exact regimen not specified	Historical longevity practice	Longevity	Consuming *Polygonatum* could promote longevity	[3,20]
*Ri Hua Zi Zhu Jia Ben Cao*	Five Dynasties period, as dated in the cited review	*Polygonatum* rhizome	Traditional use; dose not specified	Classical materia medica	Fatigue, muscle and bone weakness, and vitality	Five taxations and seven injuries, strengthening muscles and bones, reducing hunger, and supporting the spleen, stomach, heart, and lungs	[20]
*Dian Nan Ben Cao*	Ming dynasty	*Polygonatum* rhizome	Traditional use; exact dose and preparation not established here	Classical materia medica	Deficiency, fatigue, and physical vitality	Traditional uses related to replenishing deficiency and essence, supporting the spleen and stomach, and strengthening muscles and bones	[3,15]
*Zheng Lei Ben Cao*	Song dynasty materia medica compilation	*Polygonatum* rhizome	Long-term use described; dose not specified	Classical materia medica	Physical strength, complexion, hair, and teeth	*Polygonatum* with strengthening bones and marrow, increasing physical strength, improving complexion, darkening white hair, and regenerating lost teeth	[3,14]
*Sheng Ji Zong Lu*	Northern Song dynasty	*Polygonatum* rhizome	Long-term use described; exact dose not specified	Classical medical text	Qi, essence, complexion, and longevity	Traditional claims of supporting qi, consolidating essence, nourishing the lower Dantian, maintaining complexion, and promoting longevity	[3,16]
*Bao Pu Zi*	Eastern Jin period	*Polygonatum* rhizome	Long-term ingestion; the cited review mentions a ten-year period but provides no dose	Historical Daoist dietary practice	Physical strength, endurance, and longevity	Traditional claims of increased vitality, improved capacity for long-distance travel, a lighter body, and longevity	[20]
*Shi Liao Ben Cao*	Tang dynasty	*Polygonatum* as food	Dietary use; dose not specified	Traditional dietary therapy	Hunger and vitality in older people	Traditional food-related claim that consuming *Polygonatum* could alleviate hunger during old age	[20]
*Qian Jin Yao Fang:* Huangjing paste	Tang dynasty, 652 CE, as reported in the cited review	*Polygonatum*-based paste	Paste preparation; exact recipe, dose, and regimen not established here	Historical medicinal-food preparation	Complexion, hair graying, hunger, and longevity	Traditionally associated with maintaining complexion, darkening hair, reducing hunger and thirst, and promoting longevity	[20]
*Qian Jin Yi Fang:* Wujing wine	Tang dynasty, 682 CE, as reported in the cited review	*Polygonatum*-related medicinal wine	Wine preparation; ingredient ratios and dose not established here	Historical medicinal-food preparation	Liver and kidney deficiency, essence and blood, premature hair graying, and tooth looseness	Traditionally associated with nourishing essence and blood, supporting the liver and kidneys, and addressing premature hair graying and loose teeth	[20]
*Tai Ping Sheng Hui Fang:* Huangjing wine	Northern Song dynasty	*Polygonatum*-related medicinal wine	Wine preparation; dose not specified	Historical medicinal prescription	Longevity, hair graying, and tooth loss	Traditional claims of nourishing and replenishing the body, promoting longevity, darkening white hair, and regenerating lost teeth	[20]
*Tai Ping Sheng Hui Fang:* Huangjing pill	Northern Song dynasty	*Polygonatum*-containing pill	Pill preparation; exact composition and dose not established here	Historical medicinal prescription, including use in older adults	Yin deficiency, essence and marrow deficiency, and premature aging	Traditionally described as a tonic for deficiency, including in older people with insufficient essence and marrow and premature hair graying.	[20]
*Sheng Ji Zong Lu:* Erjing Wan	Song dynasty, 1117 CE, as reported in the cited review	*Polygonatum* and *Lycium barbarum*	Pill preparation; exact dose and regimen not established here	Historical medicinal prescription	Qi, essence, complexion, and longevity	Traditionally associated with consolidating essence, nourishing the lower Dantian, maintaining complexion, and promoting longevity	[16,19,20]
*Ben Cao Gang Mu:* Huangjing Cangtian wine	Ming dynasty	*Polygonatum*-containing medicinal wine	Wine preparation; exact ingredient ratios and dose not established here	Historical medicinal prescription	Muscle and bone strength, essence and marrow, and hair graying	Traditionally associated with strengthening muscles and bones, replenishing essence and marrow, and darkening white hair	[13,20]
*Tiao Ji Yin Shi Bian:* Huangjing porridge	Qing dynasty, 1813 CE, as reported in the cited review	*Polygonatum* porridge	Porridge preparation; exact proportions and dose not established here	Historical medicinal-food preparation	Deficiency and general physical weakness	Traditionally described as supporting people with deficiency-related weakness involving qi, blood, yin, or yang	[20]
Huangjing pills	clinical study	Wine-processed *Polygonatum* and *Angelica sinensis* (1:1); honey pills	Herb powder mixed with honey at 1:1.2; 9-g pills; one pill twice daily for three 1-month courses, with a 1-week break between courses	97 patients with mild-to-moderate Alzheimer’s disease; 48 in the treatment group and 49 in the control group	MMSE, HDS-R, ADL, serum SOD, GSH-Px, MDA, and IL-1*β*	The total effective rate was 91.7% versus 81.6% in the control group. The treatment group had better post-treatment cognitive and daily-living scores and higher SOD/GSH-Px activities, with lower MDA/IL-1*β* levels than the control group	[94]
Shengji Erjing granules	clinical study	*Polygonatum* and *Lycium barbarum* (10 g each in the reported formula)	5 g per dose, three times daily, for 60 days	70 patients with vascular dementia; 35 per group	MMSE, ADL, and traditional Chinese medicine syndrome scores	MMSE and ADL scores improved from baseline in the treatment group, but between-group differences were not statistically significant	[95]
Compound *Polygonatum* formula	animal experiment	*Polygonatum*, *Astragalus*, *Poria*, *Lycium barbarum*, velvet antler, and *Angelica sinensis*	Oral gavage at 2 or 4 g/kg daily for 14 days	Thirty healthy Kunming mice (10 per group); normobaric hypoxia, loaded swimming, and rotarod tests	Hypoxia survival time, loaded-swimming survival time, and rotarod endurance	The high-dose group (4 g/kg) showed longer survival under normobaric hypoxia and during loaded swimming than controls (*p* < 0.01). Both dose groups (2 and 4 g/kg) showed longer rotarod endurance (*p* < 0.01)	[96]
Compound Codonopsis polysaccharide extract	in vitro experiment	*Codonopsis pilosula*, *Poria*, and *Polygonatum* in a 3:2:1 ratio	Water extraction and alcohol precipitation; 0.25 g/L was the reported concentration with a significant effect	NIH-3T3 fibroblasts; SOD measured at 24 and 48 h	SOD activity	At 0.25 g/L, the 3:2:1 Codonopsis–*Poria*–*Polygonatum* polysaccharide preparation reduced culture-medium SOD activity versus untreated controls at 24 and 48 h (*p* < 0.01)	[97]

Abbreviations: AD, Alzheimer’s disease; ADL, activities of daily living; GSH-Px, glutathione peroxidase; HDS-R, Hasegawa Dementia Scale-Revised; MDA, malondialdehyde; MMSE, Mini-Mental State Examination; SOD, superoxide dismutase.

**Table 3:** Structural characteristics of *Polygonatum* spp. polysaccharides with reported anti-aging activities.

NO.	Name	Botanical Source	Processing, Extraction, and Purification	Monosaccharide Composition (% Molar Ratio)	Molecular Weight (Da)	Structural Characterization	Analysis Techniques	Purity And Homogeneity Assessment	Refs
1	PSP	*P. sibiricum* powder	Water extraction/reflux (10× water, 90 °C, 1 h; residue extracted at 80 °C for 2 h); 95% ethanol precipitation; chloroform: n-butanol (5:1) treatment to remove proteins; crude polysaccharide freeze-dried.	-	-	-	-	-	[27]
2	PSP-1-1	Steamed rhizome of *P. sibiricum*	Water extraction (80 °C, 2 h, three times); 80% ethanol precipitation; Sevag deproteinization; activated-carbon depigmentation; DE-52 cellulose fractionation followed by Sephadex G-200 gel filtration; desalted and lyophilized.	Man:Gal:Glc = 2.366:91.992:1.407	2.7 × 10^3^	Predominantly →4)-*β*-D-Galp-(1→ backbone (68.4%); minor →6)-Galp-(1→ (10.7%), →3)-Galp-(1→ (6.8%), and →4,6)-Galp-(1→ (8.4%), indicating branching mainly at O-6 and occasional O-3 substitution. NMR signals indicated α-and *β*-configured residues	HPGPC; PMP-HPLC; methylation-GC-MS; 1D/2D NMR; FT-IR.	Crude PSP: protein 3.2% and total sugar 95.3%. PSP-1-1 was chromatographically purified, and its Mw/dispersity was assessed	[29]
3	PSP	*P. sibiricum*	Washed and oven-dried at 60 °C for 2 days; decocted in distilled water (1:5, *w*/*v*) for 2 h; concentrated to 7 L; filtered; precipitated by adding ethanol (21 L to 7 L filtrate) and standing for 24 h; precipitate collected by filtration, concentrated in a water bath, and dried. No further purification reported.	-	-	-	-	-	[25]
4	PSP	Dried Jiu Huang Jing	Dried material powdered; refluxed with water at 80 °C for 1 h twice (10× water then 8× water); filtrates concentrated; four volumes of ethanol added for precipitation; centrifuged and freeze-dried to obtain crude PSP.	-	-	-	-	Crude polysaccharide preparation	[26]
5	PSP-NP	Processed rhizome of *P. sibiricum*	Low-molecular-weight/lipophilic compounds removed by 95% and 50% ethanol pretreatment; hot-water extraction; ethanol precipitation; neutral fraction isolated by DEAE-Sepharose FF; dialysis; further gel filtration on Sepharose 6FF; lyophilized.	Gal:Man:Glc = 68.7:24.3:7.0	43.0 × 10^3^	1,4-*β*-D-Gal, 1,4,6-*β*-D-Gal, T-*α*-D-Man, 1,4-*α*-D-Glc, and T-*α*-D-Glc	Methanolysis/GC; methylation-GC-MS; FT-IR; ^1^H-NMR and ^13^C-NMR; size-exclusion chromatography	Homogeneity and Mw assessed by size-exclusion chromatography.	[28]
6	PSP	*P. sibiricum*	-	-	-	*-*	-	Supplier-reported purity 98%	[98]
7	PSP-1	Purified fraction from *P. sibiricum* rhizome polysaccharides	Starting PSPs purified by DEAE-52 anion-exchange chromatography with NaCl elution, followed by Sephacryl-200 gel-permeation chromatography; 161.6 mg PSP-1 obtained.	-	1.8796 × 10^4^	-	UV and IR spectroscopy; DEAE-52 and Sephacryl-200 chromatography for purification; HPGPC for Mw determination.	UV spectrum showed no absorption peaks associated with nucleic acids/proteins at 260/280 nm; PDI = 1.15	[57]
8	PCP-1	*P. cyrtonema*	Water reflux (20× water, 80 °C, 2 h, twice); concentrate; 95% ethanol added to 80% final concentration at 4 °C overnight; precipitate washed with ethanol/acetone; activated carbon for depigmentation; Sevag deproteinization; DEAE-Sepharose FF followed by HW-55F gel filtration; freeze-dried.	Man:Glc:Gal:Ara = 1:2.67:0.25:0.089	3.35 × 10^3^	-	PMP-HPLC for monosaccharide composition; UV spectroscopy; DEAE-Sepharose/HW-55F for purification	No UV absorption at 260/280 nm was interpreted as absence of nucleic acids/proteins	[6]
9	PCHP	Processed rhizome of *P. cyrtonema Hua*	90% ethanol ultrasonication (250 W, 1 h) to remove lipid-soluble material; residue dried at 50 °C; water ultrasonication (250 W, 1 h) repeated three times; concentrate; 80% ethanol precipitation at 4 °C overnight; Sevag deproteinization; dialysis (7 kDa cutoff) repeated six times; lyophilized.	Man:GalA:Glu:Gal:Xyl:Ara = 13.8:3.5:22.7:2.7:1:1.3	8.77 × 10^3^–1.84 × 10^6^	-	HPSEC-RID-MALLS, HPLC	-	[30]
10	PS2-4	*P. kingianum* dried/crushed root material	80% ethanol reflux to remove small molecules; water extraction (1:30 *w*/*w*, 95 °C, 1 h, twice); 80% ethanol precipitation; fermentation with Lactobacillus casei (37 °C, 48 h); Sevag deproteinization and ethanol re-precipitation; PS2-4 obtained by sequential 100, 50, and 10 kDa ultrafiltration.	Glc:Fru:GalA:Gal:Xyl:Fuc:Ara:GlcA:Man = 69.80:18.18:4.03:2.72:1.74:1.99:0.42:0.67:0.45	10–50 × 10^3^ (ultrafiltration-defined fraction; not directly measured Mw)	-	SEC-MALS-RI, FT-IR, SEM, GC	Total sugar measured by phenol-sulfuric acid; protein by Bradford assay; uronic acid by m-hydroxydiphenyl method.	[37]
11	RPRP	Polygonati Rhizoma dried rhizome	Raw rhizome preparation. Crude polysaccharide obtained by water extraction and ethanol precipitation; protein removed by Sevag method; pigment removed with NKA-9 macroporous resin; 3500 Da dialysis; concentrated and lyophilized.	Man:Rha:Glc:Gal:Ara = 0.18:0.06:1:0.11:0.07	-	-	HPLC after acid hydrolysis and derivatization;	-	[99]
12	PPRP	Polygonati Rhizoma dried rhizome	Wine-steamed PPR prepared by adding 20% yellow wine, smothering for 6 h, stewing for 10 h followed by a further 8 h, slicing, and drying at 60 °C for 48 h. Polysaccharide extracted and purified as for RPRP: water extraction/ethanol precipitation, Sevag deproteinization, NKA-9 depigmentation, 3500 Da dialysis, lyophilization.	Man:Rha:Glc:Gal:Ara = 0.97:0.42:1:3.02:0.67	-	-	HPLC after acid hydrolysis and derivatization;	-	[99]

Abbreviations: Ara, arabinose; Fru, fructose; Fuc, fucose; Gal, galactose; GalA, galacturonic acid; Glc, glucose; GlcA, glucuronic acid; Man, mannose; Rha, rhamnose; Xyl, xylose; FT-IR, Fourier-transform infrared spectroscopy; GC-MS, gas chromatography–mass spectrometry; HPGPC, high-performance gel permeation chromatography; IR, infrared spectroscopy; PDI, polydispersity index; PMAA, partially methylated alditol acetate(s); UV, ultraviolet spectroscopy; HPLC, high-performance liquid chromatography; HPLC-RID, high-performance liquid chromatography with refractive index detection; HPSEC-RID-MALLS, high-performance size-exclusion chromatography with refractive index detection and multi-angle laser light scattering; NMR, nuclear magnetic resonance; PMP, 1-phenyl-3-methyl-5-pyrazolone; PMP-HPLC, PMP derivatization followed by high-performance liquid chromatography; SEC-MALLS-RI, size-exclusion chromatography coupled with multi-angle laser light scattering and refractive index detection; SEM, scanning electron microscopy; UHPLC-MS/MS, ultra-high-performance liquid chromatography–tandem mass spectrometry.

**Table 4:** Reported anti-aging activities and associated mechanisms of *Polygonatum* spp. polysaccharides and related preparations (“↓”, decrease; “↑”, increase).

Sources	Compound Name	Types	Study Model/Evidence Category	Specific Pathways/Targets	Doses/Duration	Effects/Mechanisms	Evidence Level for Mechanism	Refs
**Antioxidant**								
*P. sibiricum*	PSP	In vivo	Chemically induced aging model: D-gal-induced cardiac aging mouse model	Oxidative-stress-associated markers	200 and 400 mg/kg/day for 60 days	ROS, MDA, 8-OHdG, 4-HNE, p53, p21, CK, cTnT ↓; SOD ↑	Biomarker association	[8]
*P. sibiricum*	PSP	In vivo	Chemically induced aging model: D-gal-induced aging rat model	-	100 mg/kg/day for 35 days	Klotho mRNA/protein, SOD, GSH-Px ↑; MDA, NO, NOS ↓	Biomarker association	[98]
*P. sibiricum*	PSP-1-1	In vivo	Lifespan/healthspan study: *C. elegans*	Glycolysis-associated changes	100 μg/mL	Median lifespan ↑ 29.88%; Aβ-induced paralysis delayed (PT50 ↑ 51.42%); oxidative-stress resistance ↑; ROS ↓ 21.36%	Biomarker association	[29]
*P. cyrtonema*	PCP-1	In vivo	Lifespan/healthspan study: *C. elegans*	IIS pathway-associated markers; causal involvement not genetically validated	0.5, 1, 2 mg/mL; lifespan monitored until death	Lifespan ↑ up to 20.16% (1 mg/mL); DAF-16/FOXO nuclear translocation; SOD/CAT activities ↑; expression of *daf-16*, *skn-1*, *sod-3* and *hsp-16.2* ↑; *daf-2* and *age-1* expression, ROS, MDA and lipofuscin ↓	Biomarker association	[6]
*P. cyrtonema*	PCHP	In vivo	Lifespan/healthspan study: *C. elegans*	DAF-16/SIR-2.1/SKN-1	5.0 mg/mL; lifespan monitored until death; ROS and lipofuscin assessed on day 8 of adulthood	Lifespan ↑ 15.78% (5.0 mg/mL); motility and stress resistance ↑; ROS and lipofuscin ↓	Genetic validation	[30]
*P. kingianum*	PS2-4	In vitro	In vitro antioxidant assay (not a direct aging model): *DPPH radical-scavenging and Fe^3+^-reducing assays*	Antioxidant capacity assays	0.1 mg/mL	DPPH radical-scavenging capacity and Fe^3+^-reducing power ↑	Not applicable: antioxidant assay only	[37]
*P. kingianum*	PS2-4	In vivo	Lifespan/healthspan study: *C. elegans*	Lifespan/healthspan outcomes; no pathway tested	0.5 mg/mL; lifespan monitored until death; movement and lipofuscin assessed during adult aging	Lifespan ↑ 20.70%; movement ability improved; lipofuscin accumulation ↓	Not applicable: phenotypic lifespan/healthspan outcomes; no pathway tested	[37]
*P. kingianum*	PKP-1b	In vivo	Lifespan/healthspan study: *C. elegans*; chemically induced aging model: D-gal-induced C57BL/6J mice	IIS–DAF-16/FOXO pathway-associated changes	*C. elegans*: tested across several concentrations; mouse: 200 mg/kg/day orally, 8 weeks	*C. elegans* lifespan and motor function ↑; lipofuscin ↓; D-gal mouse neurobehavioral and oxidative-damage endpoints improved; DAF-16 nuclear localization and SOD-3 reporter activity ↑	Biomarker association	[100]
Polygonati Rhizoma	PRP	In vivo	Lifespan/healthspan study: *C. elegans* N2; AD-like phenotype: A*β*-expressing CL4176 worms	Senescence-, autophagy- and mitochondrial-related transcriptomic signatures; phenylalanine/purine metabolomic associations	12.5, 50, 200, 800 μg/mL (lifespan assay); maximum lifespan response reported at 200 μg/mL	N2 lifespan ↑ (maximum lifespan ↑ ~47.41% at 200 μg/mL); lipofuscin and ROS ↓; locomotion ↑; Aβ-associated paralysis delayed	Biomarker association	[101]
Polygonati Rhizoma	PRP	In vivo	Lifespan/healthspan study: *C. elegans*	IIS pathway	2 mg/mL	Average lifespan ↑ 10.44%; *daf-2* expression ↓; *daf-16* and *sod-3* expression ↑	Genetic validation (daf-16 mutant worms)	[99]
**Regulation of gut microbiota**								
*P. sibiricum*	PSP	In vivo	Chemically induced aging model: D-gal-induced aging mouse model	Microbiota–gut–brain axis	150, 300, 600 mg/kg/day	Distance in target zone and platform crossings ↑; Bacteroidota ↑; Firmicutes, Patescibacteria, Actinobacteriota, Campilobacterota ↓	Biomarker association	[27]
*P. sibiricum*	PSP-1-1	In vivo	Lifespan/healthspan study: *C. elegans*	Glycolysis-associated changes; causal involvement not established	100 μg/mL	A*β* oligomers ↓; glycolysis-related genes (*pfk-1.1*, *gpi-1*, *aldo-1*, *aldo-2*, *pyl-1*) ↑	Biomarker association	[29]
*P. sibiricum*	PSP-1-1	In vitro	In vitro human gut microbiota fermentation	Gut microbiota composition, SCFA production and predicted microbial carbohydrate-metabolism pathways	-	Firmicutes and SCFA-producing bacteria (e.g., Lactobacilli) ↑; Proteobacteria ↓; acetate, propionate and butyrate ↑	Biomarker association	[29]
*P. sibiricum*	PSP	In vivo	Chemically induced aging model: D-gal-induced aging male C57BL/6J mice	-	150 or 600 mg/kg/day for 4 weeks (PSP treatment; 8-week model)	*Lactobacillus, Bifidobacterium and Alloprevotella ↑; Firmicutes/Bacteroidota* ratio partially restored (not statistically significant)	Biomarker association	[26]
*P. sibiricum*	PSP	In vivo/in vitro	Naturally aged animal model: 16-month-old C57BL/6J mice; cell-based stress/senescence model: D-gal-treated Caco-2 cells	TLR4/NF-κB pathway and gut microbiota–metabolite changes; intestinal barrier integrity	Mice: 200 or 400 mg/kg/day orally for 8 weeks; Caco-2: 100, 200, 400 μg/mL	Intestinal permeability and pro-inflammatory cytokines ↓; tight-junction integrity ↑; microbial/metabolite profiles altered; TLR4 agonist RS09 attenuated cellular protection	Pharmacological perturbation	[102]
*P. sibiricum*	PSP-NP	In vivo	Naturally aged animal model: aged male SPF C57BL/6Cnc mice	-	50, 100, 200 mg/kg/day for 6 weeks	*Bifidobacterium* and *Lactobacillus* counts, acetate, propionate and butyrate ↑; Firmicutes/Bacteroidetes balance improved; sIgA secretion and jejunal Mucin 2 mRNA ↑; *Escherichia coli* counts ↓ (not significant)	Biomarker association	[28]
*P. kingianum*	PS2-4	In vivo	Aging-related physiological outcomes: *C. elegans* (adult days 2, 7 and 15); pathogen-stress challenge	UPRmt–ATFS-1/hsp-60; mitochondrial function and intestinal barrier homeostasis	0.5 mg/mL; measurements at adult days 2, 7 and 15	ATP and mitochondrial respiratory-chain markers ↑; ROS and age-related intestinal leakage ↓; stress-induced hsp-60 response ↑; ATFS-1 RNAi abolished the PS2-4 enhancement of hsp-60 response	Genetic validation	[103]
*P. sibiricum*	PSP-1	In vivo	Age-related disease model: 5 × FAD Alzheimer’s disease mice	-	30 mg/kg/day for 3 months	*Akkermansia muciniphila*, *Firmicutes/Bacteroidetes* balance, occludin/ZO-1 *↑*; *Helicobacter typhlonius, H. mastomyrinus*, intestinal permeability, colonic A*β* deposition *↓*	Biomarker association	[57]
**Regulation of mitochondrial dysfunction**								
*P. sibiricum*	PSP	In vitro	Cell-based senescence model: D-gal-induced senescence in C2C12 myoblasts	PI3K/Akt/mTOR pathway	0.6/1.2 mg/mL for 48 h	*p*-PI3K, *p*-Akt, *p*-mTOR, MyoD, Myogenin, MyHC ↑; senescence markers (p16, p21, p53), MuRF1, Atrogin-1 ↓	Pharmacological inhibition	[25]
		In vivo	Chemically induced aging model: D-gal-induced skeletal-muscle aging in male C57BL/6J mice		60/120 mg/kg for 8 weeks		Biomarker association	
*P. sibiricum*	PSP	In vitro	Cell-based senescence model: D-gal-induced senescence in C2C12 myotubes	MAM/Ca^2+^-homeostasis-associated changes	50/100 mg/L for 24 h	SIRT1/SIRT3, mitochondrial function (ATP/OCR/MMP) ↑; MAM formation, Ca^2+^ overload, senescence markers (p16, p53)↓	Biomarker association	[45]
		In vivo	Naturally aged animal model: aged C57BL/6J mice		200 mg/kg/day for 4 weeks		Biomarker association	
**Regulation of metabolism**								
*P. sibiricum*	PSP	In vivo	Chemically induced aging model: D-gal-induced aging mice	-	150 or 600 mg/kg/day for 4 weeks (PSP treatment; 8-week model)	Octadecanedioic acid ↓ in the model vs. control and ↑ after PSP treatment, consistent with partial reversal of aging-associated metabolomic changes	Biomarker association	[26]
*P. sibiricum*	PSP	In vivo	Chemically induced aging model: D-gal-induced aging mice	-	150, 300, 600 mg/kg/day for 8 weeks	12 significantly differential metabolites associated with PSP treatment; pathways included purine, taurine/hypotaurine, glycerophospholipid, alanine/aspartate/glutamate and riboflavin metabolism; profile shifted toward control	Biomarker association	[27]
*P. odoratum*	POP	In vivo	Chemically induced aging model: D-gal-induced accelerated aging rat model	Nrf2-mediated ferroptosis/iron-metabolism pathway	200, 400, 800 mg/kg for 12 weeks	Nrf2, FPN, FTH, GPX4, SLC7A11 and GSH ↑; ACSL4, Fe^2+^ and MDA ↓; cognitive dysfunction improved	Pharmacological Nrf2 inhibition	[59]
*P. sibiricum*	PSP	In vivo	Chemically induced aging model: D-gal-induced aging rat model	Klotho–FGF-23 axis and Akt/FOXO3a-associated marker changes	100 mg/kg/day for 35 days	Serum Ca^2+^ and Klotho mRNA/protein ↑; ALP, phosphate and FGF-23 protein ↓	Biomarker association	[98]
*P. sibiricum*	PSP	In vivo	Chemically induced aging model: D-gal-induced aging model	Metabolomic pathway associations	150, 300, 600 mg/kg/day for 4 weeks	PSP partly reversed aging-associated metabolite changes; reported biomarkers included AMP, tryptophan and 5-hydroxytryptophan	Biomarker association	[104]
**Regulate immunity**								
*P. sibiricum*	PSP-NP	In vivo	Naturally aged animal model: Aged male C57BL/6Cnc mice	Mucosal immunity	50, 100, 200 mg/kg/day for 6 weeks	sIgA ↑ in jejunum and colon; Mucin 2 mRNA ↑ in jejunum (no significant effect in colon)	Biomarker association	[28]
**Anti-inflammatory**								
*P. sibiricum*	PSP-NP	In vivo	Naturally aged animal model: Aged male C57BL/6Cnc mice	Pro-inflammatory cytokines	50, 100, 200 mg/kg/day for 6 weeks	IL-1*β* IL-6, IL-12A, IL-17, IL-23, TNF-*α ↓*; IL-4, IL-10, TGF-*β ↑*	Biomarker association	[28]
*P. sibiricum*	PSP	In vitro	Cell-based senescence model: tert-butyl hydroperoxide-induced senescence in primary rat BMSCs	NCOA4-mediated ferritinophagy; mitochondrial dysfunction and mtDNA–cGAS/STING signaling	50, 100, 200 μg/mL PSP for 24 h; TBH (100 μM) for 48 h	SA-β-gal-positive cells, intracellular Fe^2+^, mtROS and cytosolic mtDNA ↓; ATP, mitochondrial membrane potential and osteogenic differentiation ↑; NCOA4 overexpression partly reversed PSP protection	Genetic overexpression/rescue	[105]
*P. sibiricum*	PSP0/PSP5/PSP9	In vivo	Chemically induced aging model: D-gal-induced aging mouse model	-	400 mg/kg/day for 60 days	IL-1β, TNF-α, NLRP3 ↓; GluA1/2, GluN2A/2B, PSD-95 and Arc ↑	Biomarker association	[106]
*P. kingianum*	PS2-4	In vitro	Cell-based replicative-senescence model: MRC-5 replicative senescence	-	0.5 mg/mL for 48 h	Cell viability ↑; *β*-galactosidase activity ↓; secretion of SASP factors IL-6, IL-8, CXCL-10 and HGF ↓	Biomarker association	[37]
**Other mechanisms**								
*P. sibiricum*	PSP	In vivo	Chemically induced aging model: D-gal-induced brain ageing model	-	200, 400 mg/kg/day for 60 days	STXBP1, SNX8, TSC1 and Sirt3 ↑; Tcf4, TRPM2 and Numb ↓; transcriptomic changes associated with PSP treatment	Biomarker association	[58]
*P. sibiricum*	PS preparation (not isolated PSP)	In vivo	Naturally aged animal model: naturally aged Sprague–Dawley rats	-	0.33 and 1.33 g/kg (calculated from feed intake) for 5 months	BDNF expression, TrkB phosphorylation, PSD-95, synaptophysin ↑	Biomarker association	[107]

Abbreviations: 4-HNE, 4-hydroxy-2-nonenal; 5 × FAD, mouse model carrying five familial Alzheimer’s disease mutations; 8-OHdG, 8-hydroxy-2′-deoxyguanosine; A*β*, amyloid-*β*; ACSL4, acyl-CoA synthetase long-chain family member 4; Akt, protein kinase B; ALP, alkaline phosphatase; AMP, adenosine monophosphate; AMPK, AMP-activated protein kinase; ATFS-1, activating transcription factor associated with stress-1; ATP, adenosine triphosphate; BDNF, brain-derived neurotrophic factor; BMSCs, bone marrow mesenchymal stem cells; *C. elegans*, *Caenorhabditis elegans*; CAT, catalase; cGAS, cyclic GMP–AMP synthase; CK, creatine kinase; cTnT, cardiac troponin T; D-gal, D-galactose; DAF, defective in dauer formation; DAF-16, dauer formation abnormality 16; DPPH, 2,2-diphenyl-1-picrylhydrazyl; FAC, ferric ammonium citrate; FGF-23, fibroblast growth factor 23; FOXO, forkhead box O; FOXO3a, forkhead box O3a; FPN, ferroportin 1; FTH, ferritin heavy chain; GluA1/2, AMPA-type glutamate receptor subunits 1 and 2; GluN2A/2B, NMDA receptor subunits 2A and 2B; GPX4, glutathione peroxidase 4; GSH, reduced glutathione; GSH-Px, glutathione peroxidase; HGF, hepatocyte growth factor; HO-1, heme oxygenase 1; Hsp-16.2, heat shock protein 16.2; IIS, insulin/insulin-like growth factor signaling; IL, interleukin; MAM, mitochondria-associated membrane; MAPK, mitogen-activated protein kinase; MDA, malondialdehyde; MMP, mitochondrial membrane potential; MRC-5, human fetal lung fibroblast cell line; mtDNA, mitochondrial DNA; mtROS, mitochondrial reactive oxygen species; MuRF1, muscle RING finger 1; MyHC, myosin heavy chain; MyoD, myogenic differentiation 1; NCOA4, nuclear receptor coactivator 4; NF-κB, nuclear factor kappa B; NLRP3, NLR family pyrin domain-containing 3; NO, nitric oxide; NOS, nitric oxide synthase; Nrf2, nuclear factor erythroid 2-related factor 2; OCR, oxygen consumption rate; *p*-Akt, phosphorylated Akt; *p*-mTOR, phosphorylated mechanistic target of rapamycin; *p*-PI3K, phosphorylated phosphoinositide 3-kinase; PCHP, *P. cyrtonema* Hua polysaccharides; PCP-1, purified polysaccharide fraction from *P. cyrtonema*; PGC-1α, peroxisome proliferator-activated receptor gamma coactivator 1 alpha; PI3K, phosphoinositide 3-kinase; PKP-1b, purified agavin-type fructan fraction from *P. kingianum*; POP, *P. odoratum* polysaccharide (YuZhu); PRP, Polygonati Rhizoma polysaccharide; PS, *Polygonatum sibiricum* preparation; PS2-4, low-molecular-weight fraction of fermented *P. kingianum* polysaccharides; PSD-95, postsynaptic density protein 95; PSP(s), *Polygonatum* species polysaccharide(s); in individual studies, PSP denotes the preparation as named by the original authors; PSP-1, monomeric polysaccharide from *P. sibiricum*; PSP-1-1, low-molecular-weight polysaccharide fraction from steamed *P. sibiricum*; PSP-NP, neutral polysaccharide from *P. sibiricum*; PSP0/PSP5/PSP9, *P. sibiricum* polysaccharide preparations after 0, 5 and 9 steaming cycles; PT50, time to 50% paralysis; ROS, reactive oxygen species; RS09, TLR4 agonist; RNAi, RNA interference; SA-*β*-gal, senescence-associated *β*-galactosidase; SASP, senescence-associated secretory phenotype; SCFA(s), short-chain fatty acid(s); sIgA, secretory immunoglobulin A; SIR-2.1, sirtuin 2.1; SIRT1/SIRT3, sirtuin 1/sirtuin 3; SLC7A11, solute carrier family 7 member 11; SOD, superoxide dismutase; SPF, specific pathogen-free; STING, stimulator of interferon genes; TBH, tert-butyl hydroperoxide; TGF-β, transforming growth factor beta; TLR4, Toll-like receptor 4; TNF-α, tumor necrosis factor-alpha; TrkB, tropomyosin receptor kinase B; UPRmt, mitochondrial unfolded protein response; ZO-1, zonula occludens-1.

## Data Availability

No new data were created or analyzed in this study. Data sharing is not applicable to this article.
